# Hybrid superhydrophilic–superhydrophobic micro/nanostructures fabricated by femtosecond laser-induced forward transfer for sub-femtomolar Raman detection

**DOI:** 10.1038/s41378-019-0090-1

**Published:** 2019-09-23

**Authors:** Xiaodan Ma, Lan Jiang, Xiaowei Li, Bohong Li, Ji Huang, Jiaxing Sun, Zhi Wang, Zhijie Xu, Liangti Qu, Yongfeng Lu, Tianhong Cui

**Affiliations:** 1 Laser Micro/Nano Fabrication Laboratory, School of Mechanical Engineering, Beijing Institute of Technology, 100081 Beijing, China; 2 Department of Mechanical Engineering, Tsinghua University, 100084 Beijing, China; 3 School of Chemistry and Chemical Engineering, Beijing Institute of Technology, 100081 Beijing, China; 4 Department of Electrical and Computer Engineering, University of Nebraska-Lincoln, Lincoln, NE 68588-0511 USA; 5 Department of Mechanical Engineering, University of Minnesota, Minneapolis, MN 55455 USA

**Keywords:** Sensors, Nanofabrication and nanopatterning

## Abstract

Raman spectroscopy plays a crucial role in biochemical analysis. Recently, superhydrophobic surface-enhanced Raman scattering (SERS) substrates have enhanced detection limits by concentrating target molecules into small areas. However, due to the wet transition phenomenon, further reduction of the droplet contact area is prevented, and the detection limit is restricted. This paper proposes a simple method involving femtosecond laser-induced forward transfer for preparing a hybrid superhydrophilic–superhydrophobic SERS (HS-SERS) substrate by introducing a superhydrophilic pattern to promote the target molecules to concentrate on it for ultratrace detection. Furthermore, the HS-SERS substrate is heated to promote a smaller concentrated area. The water vapor film formed by the contact of the solution with the substrate overcomes droplet collapse, and the target molecules are completely concentrated into the superhydrophilic region without loss during evaporation. Finally, the concentrated region is successfully reduced, and the detection limit is enhanced. The HS-SERS substrate achieved a final contact area of 0.013 mm^2^, a 12.1-fold decrease from the unheated case. The reduction of the contact area led to a detection limit concentration as low as 10^−16^ M for a Rhodamine 6G solution. In addition, the HS-SERS substrate accurately controlled the size of the concentrated areas through the superhydrophilic pattern, which can be attributed to the favorable repeatability of the droplet concentration results. In addition, the preparation method is flexible and has the potential for fluid mixing, fluid transport, and biochemical sensors, etc.

## Femtosecond laser processing for sub-femtomolar Raman analysis

A simple, flexible femtosecond laser process yields substrates that enable the detection of miniscule traces of target molecules with surface-enhanced Raman scattering (SERS). SERS is a powerful tool for performing analysis of samples such as food and biological specimens, but detecting ultra-scarce molecules has proven to be a challenge. Lan Jiang of the Beijing Institute of Technology and his colleagues have now developed a strategy for concentrating samples to such an extent that even sub-femtomolar quantities can be accurately detected with SERS. They created silicon substrates layered with a superhydrophobic polymer, and then used a femtosecond pulsed laser to prepare patterns in that layer that leave an exposed superhydrophilic surface. Dilute samples applied to the substrate subsequently become highly concentrated within those patterned sites, enabling straightforward SERS analysis with minimal effort.

## Introduction

Ultratrace detection plays a crucial role in chemical and biological analyses for applications such as food safety testing^1^, drug testing^2^, explosives testing^3^, and early diagnosis^4^. Raman spectroscopy is considered one of the most promising analytical methods because it provides fingerprint information on target molecules and has high sensitivity^5–7^. It has been extensively used for practical analysis and detection of properties of compounds such as Rhodamine 6G (R6G)^8^, glucose^9^, and tyrosine solution^10^.

In addition to the electromagnetic field effect, the Raman scattering signal intensity has been confirmed to be as relevant as the analyte molecule concentration^11,12^. In the detection of highly diluted solutions whose concentrations are on the picomolar or femtomolar level, the target molecules are too dispersed to be detected, which limits the Raman detection sensitivity. Therefore, concentrating target molecules into a small area without loss to increase the concentration of the analyte molecules is the key challenge in enhancing Raman detection sensitivity. Recent research has reported a superhydrophobic surface-enhanced Raman scattering (S-SERS) substrate, which has been considered an effective strategy to solve the aforementioned problems^13–17^. When an S-SERS substrate is used, the concentration of the target molecule can be improved by increasing the contact angle and reducing the adhesion of the substrate to obtain a higher sensitivity. Wang et al. reported the fabrication of an S-SERS substrate by using a femtosecond laser^18^; the minimum area of this substrate after evaporation was reduced to 0.14 mm^2^, and the detection limit was 10^−14^ M. Reducing the concentrated area was confirmed to enhance the Raman signal. However, during droplet concentration, the contact angle suddenly changes at a certain moment and then sharply decreases due to the wet transition phenomenon^19^, which further prevents the reduction of the droplet contact area. In addition to higher sensitivity, an efficient and perfect SERS substrate should achieve stable results. Applying S-SERS substrates in practical applications is difficult because of the uncertainty regarding the final concentration position and the unstable concentrated area size; for example, locating the target molecule and obtaining stable signals is difficult in practical applications. Hence, controlling and reducing the final droplet concentrated area size remains a considerable challenge in the use of S-SERS substrates.

Research groups have recently prepared special wettability SERS (SW-SERS) substrates by introducing a differential wettability region, which has successfully concentrated droplets into a relatively small area and enhanced detection limits. Song et al. applied lithography and vapor phase deposition to prepare a gradient superhydrophobic surface on silicon, and the detection limit was 10^−15^ M^20^. Li et al. prepared a micropatterned superhydrophilic Au-areole array on a silicon substrate for SERS detection using a multistep process that involved metal-assisted chemical etching, surface fluorination treatment, partial removal using ultraviolet light, and electrochemical deposition. The detection limit was 10^−15^ M^21^. However, the processing methods used above are multistep and require complex equipment or operations. A simple preparation method is still a challenge for SW-SERS substrates.

Compared with the aforementioned methods, a femtosecond laser is easy to operate and has a wide processing range; based on this, it has a good application potential for the preparation of micro/nanostructure surfaces^22–28^. Femtosecond laser-induced forward transfer (FLIFT) can easily realize the combination of intrinsic hydrophilic materials and intrinsic hydrophobic materials, and it is a simpler and more efficient method for preparing special wettability surface structures without additional surface modification. Furthermore, because of the Leidenfrost effect^29,30^, when the substrate is heated at a high temperature, the droplets in contact with the substrate evaporate to form a water vapor film. The water vapor film maintains the droplets in a spherical shape to enhance their stability; thus, the droplets do not collapse as a result of the wet transition during the concentration process, which enables concentration of the droplet into smaller spheres to enhance the detection limit. In addition, heating can accelerate droplet evaporation, which considerably shortens the droplet concentration time and improves the detection efficiency of the HS-SERS substrate. However, the water vapor film causes the droplets to roll off the substrate. A superhydrophilic pattern can effectively solve this problem by capturing the droplets and concentrating them in designated areas.

In summary, developing a simple method for preparing a SERS substrate that can achieve a small droplet concentration area and stable and controllable results is required. Accordingly, this study proposes a simple and efficient method involving the use of FLIFT for preparing an HS-SERS substrate for ultratrace detection that can achieve directional concentration of droplets from superhydrophobic regions to a superhydrophilic pattern without loss. The method used directly achieves the combination of two surfaces with extreme wettability by a femtosecond laser without additional surface modification. First, polydimethylsiloxane (PDMS) was sputtered onto the surface of silicon through FLIFT to form a periodic superhydrophobic structure. Subsequently, a femtosecond laser was applied to selectively remove the superhydrophobic structure while ablating the underlying silicon substrate to form a superhydrophilic pattern. We mixed the target molecules and Au nanoparticles and then concentrated them into the pattern through heating to achieve ultratrace detection. The HS-SERS substrate achieved a final contact area of 0.013 mm^2^, 12.1 times less than the unheated case. The reduction in the contact area led to a detection limit concentration as low as 10^−16^ M for the R6G solution. In addition, the HS-SERS substrate accurately controlled the concentrated area size through the superhydrophilic pattern because of the favorable repeatability of the droplet concentration results.

## Materials and methods

**Fig. 1:**
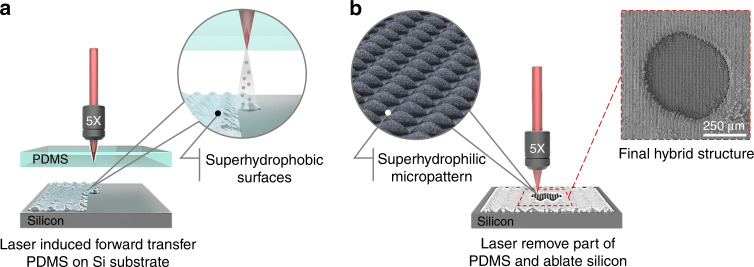
Schematic of the fabrication process of the HS-SERS substrate. **a** First, PDMS was sputtered through FLIFT on the silicon surface to form a superhydrophobic surface (inset). **b** In the second step, the femtosecond laser was used for selective removal of the jet to obtain a silicon patterned superhydrophilic surface (inset). The SEM image of the final hybrid superhydrophilic–superhydrophobic structures is shown as the inset picture with a red dotted line

The FLIFT method used in this study requires the laser to be able to pass through the donor, the donor material be hydrophobic, and the receiver material be hydrophilic. Based on these requirements, silicon (crystal orientation: 111) was used as the receiver because of its intrinsically hydrophilic nature. PDMS was used as the donor because of its intrinsic hydrophobicity, good light transmission, and good biocompatibility. We used a femtosecond laser beam with a width of 35 fs, repetition rate of 1000 Hz, and translating speed of 1000 μ ms^−1^. Figure 1 presents a schematic of the HS-SERS substrate fabrication process, which involved two steps in situ. First shown in Fig. 1a, PDMS was sputtered through FLIFT on the silicon surface to form a superhydrophobic surface. The donor PDMS and the receiver silicon were tightly fitted together with a gap of 20 μm. A femtosecond laser was focused 20 μm above the lower surface of the PDMS using a 5× microscope objective lens (NA = 0.15), and the laser ablation scanning pitch was controlled using a six-dimensional translation stage. Second, shown in Fig. 1b, PDMS was removed, and without changing the machining position, the femtosecond laser was directly focused on the silicon surface, followed by selective removal of the PDMS while simultaneously ablating the silicon surface to form a superhydrophilic pattern with a convex morphology. The final hybrid superhydrophilic–superhydrophobic structure is shown as the SEM illustration with a red dotted line. In summary, our proposed processing method is a very simple technique for obtaining an HS surface.

## Results and discussion

### Femtosecond laser processing

**Fig. 2:**
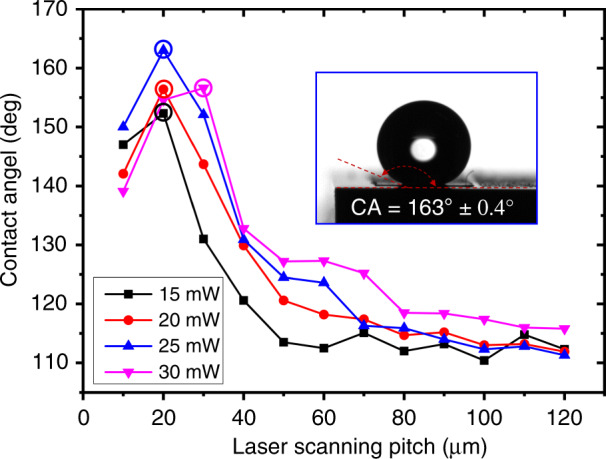
Contact angles of the fabricated superhydrophobic surfaces without a superhydrophilic micropattern at different laser powers (15–30 mW) and scanning pitches (10–120 μm); circles in different colors indicate the optimal contact angles for different laser powers. The volume of the droplets used was 2 μL

**Fig. 3:**
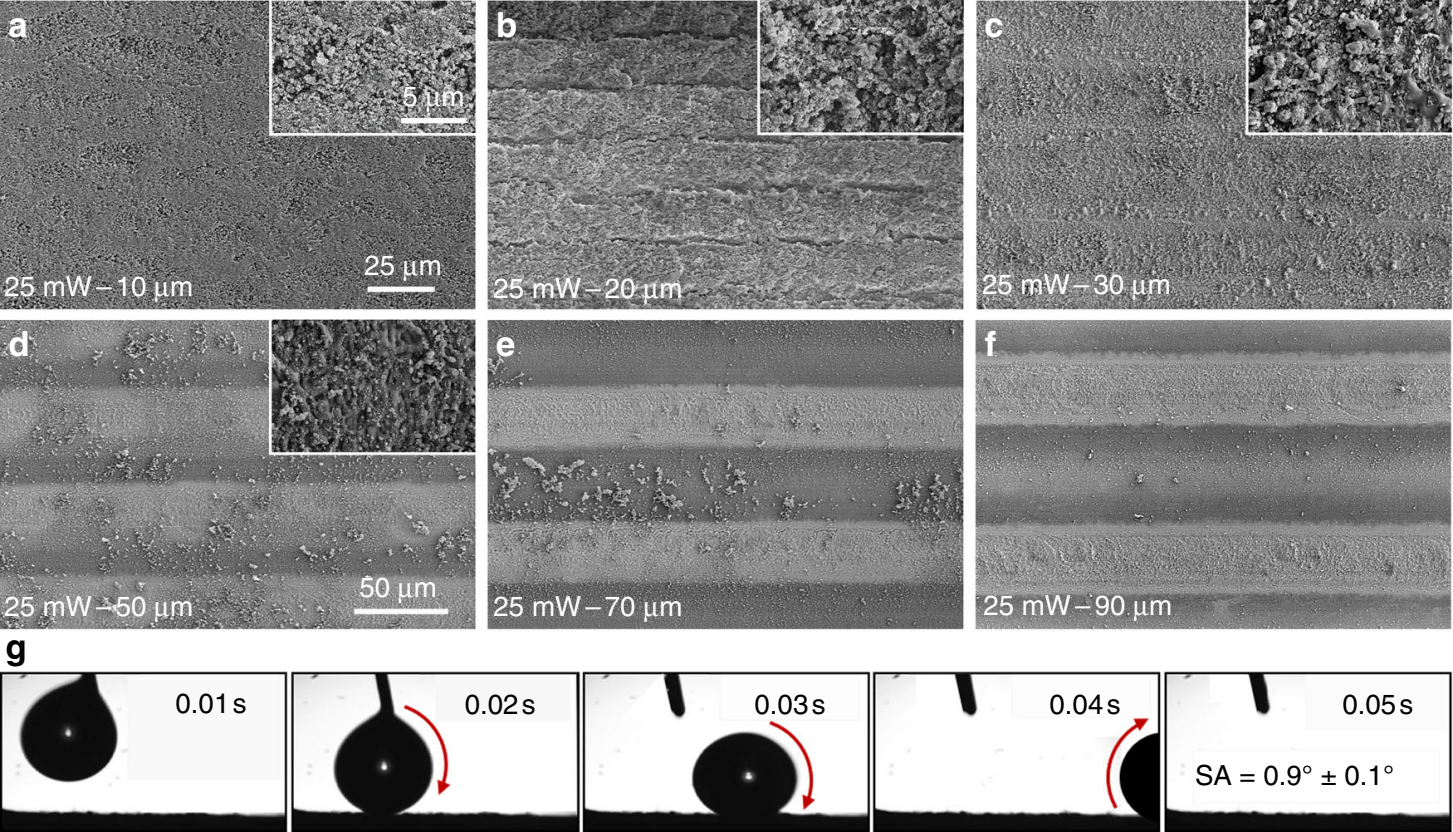
SEM images of morphologies at different laser scanning pitches and a laser power of 25 mW: **a** 10 μm, **b** 20 μm, **c** 30 μm, **d** 50 μm, **e** 70 μm, and **f** 90 μm; **b**, **c** share the same scale bar with **a**, which is 25 μm, and the inset is a magnified SEM image with a scale bar of 5 μm; **d**–**f** share the same scale bar with **d**, which is 50 μm. **g** Snapshot of water droplets rolling on the superhydrophobic surfaces with a tilt angle of less than 1°

The superhydrophobic surface produced in the first step was applied to determine the droplet concentration effect. Figure 2 shows the effect of the laser scanning pitch and laser power on the change in the contact angle; the volume of the droplets used was 2 μL. The scanning pitch was changed from 10 to 120 μm, and the laser power was changed from 15 to 30 mW. When the laser power increased, the optimal contact angle (denoted by the different colored circles) first increased and then decreased, and the contact angles observed at the various laser powers all exceeded 150°, demonstrating the creation of a superhydrophobic surface. The maximum contact angle (163° ± 0.4°, as shown in the inset) was achieved at a laser power of 25 mW and a scanning pitch of 20 μm. Figure 2 also shows that as the scanning pitch increased, the contact angle first increased and then declined. Consider, for example, the surface prepared at a laser power of 25 mW. Figure 3a–f depicts the scanning electron microscopy (SEM) images of the surface morphologies observed at different laser scanning pitches; Fig. 3b presents the optimal scanning pitch with the largest contact angle. When the scanning pitch was too small, the overlap ratio of the laser processing area was high, and PDMS was peeled off from the depth of focus. At this time, the height of the superhydrophobic structure was 9 μm. When the scanning pitch was increased, the overlap ratio of the laser processing area was insufficient to cause the PDMS to peel off. The height of the superhydrophobic structure was equal to that of the jet accumulation at 800 nm (Fig. S1).

When the laser scanning pitch increased from 10 to 20 μm (Fig. 3a, b), the superhydrophobic structure height was 9 μm. This phenomenon can be explained by the Cassie–Baxter model^31^, which is described as follows:

1 $$\cos \theta _{{\mathrm{CA}}} = f\cos \theta - \left( {1 - f} \right),$$

where *θ*_CA_ and *θ* are the Cassie–Baxter contact angle and Young contact angle, respectively, and *f* is the surface fraction of the surface contacted by the droplets occupying the area of the entire surface. When the scanning pitch was increased, the contact area of the droplets with air increased (Fig. S2), resulting in a smaller ratio of the solid contact area to the entire contact area, and *f* decreased; therefore, the contact angle eventually increased. When the laser scanning pitch continued to increase from 20 to 30 μm (Fig. 3a, b), a PDMS thickness of 1 μm was observed (Fig. S2), which was much smaller than the laser scanning pitch, and the air retention height below the droplet was less than the PDMS height; at this moment, the droplet contact model was transitioning from the Cassie–Baxter model to the Wenzel model^32^, which is described as follows:

2 $$\cos \theta _{\mathrm{W}} = r\cos \theta,$$

where *θ*_W_ is the Wenzel contact angle and *r* is the roughness factor, defined as the ratio of the true surface area to the projected area. When the scanning pitch continued to increase, the ratio of the area occupied by the untreated silicon substrate increased and *r* decreased (Fig. 3d–f), causing the contact angle to continue to decrease. In addition, the intrinsic hydrophilicity of the silicon substrate contributed to the reduction in the contact angle. In summary, the contact angle of the PDMS structure produced using FLIFT first increased and then decreased with the increase in the scanning pitch, and this tendency applied to not only a laser power of 25 mW but also other power conditions.

Figure 3g presents the snapshot of water droplets rolling on the structure in Fig. 3b. It was very easy for a 5-μL water droplet to roll away even when the tilt angle was only 1° and the structure was processed with a laser power of 25 mW and scanning pitch of 20 μm. This proves that the superhydrophobic surface produced in this study resembled a lotus leaf surface with ultralow adhesion. This structure had an ultrahigh hydrophobic angle and ultralow adhesion, which were determined as subsequent processing parameters, and hence, the structure met our subsequent use requirements.

**Fig. 4:**
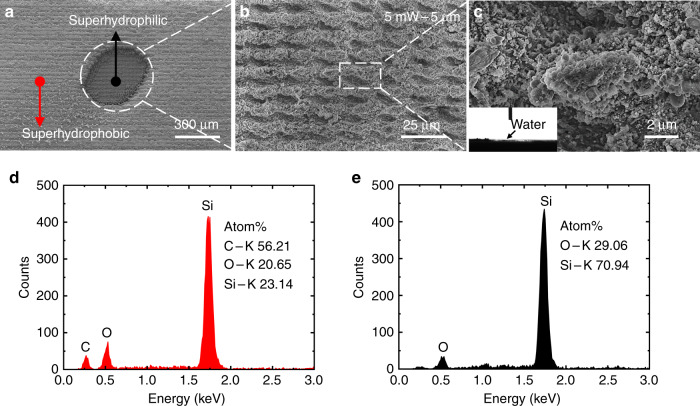
Preparation and characterization of superhydrophilic patterns. **a** SEM image of the HS surface with a superhydrophilic pattern with a diameter of 300 μm (dotted circle). **b** SEM image of the superhydrophilic silicon surface fabricated using a femtosecond laser with a laser power of 5 mW and scanning pitch of 5 μm. **c** Further enlarged SEM image of **b**; the illustration shows the contact angle of superhydrophilic surfaces at this laser power. **d** EDX results for the superhydrophobic surface of the HS-SERS substrate. **e** EDX results of the patterned superhydrophilic regions of the HS-SERS substrate

After the superhydrophobic surface was prepared, a superhydrophilic micropattern on silicon was achieved using a femtosecond laser operated at a low laser power of 5 mW and scanning pitch of 5 μm. A micropatterned convex structure with superhydrophilic properties was processed on the silicon surface, and the femtosecond laser was used to remove the local periodic PDMS structure. Figure 4a depicts the SEM image of the HS surface with a pattern diameter of 300 μm (dotted circle). The area indicated by the red arrow represents the superhydrophobic surface and that indicated by the black arrow represents the superhydrophilic pattern. A partially magnified SEM image is shown in Fig. 4b, and a further enlarged SEM image is shown in Fig. 4c. A 2-μL droplet was dropped on the processed silicon surface with a large convex structure area, on which the droplet spread rapidly. The lateral images of the droplet captured using a lateral imaging system are presented in the inset of Fig. 4c. We determined that the contact angle of the silicon surface with a convex structure was 0°, demonstrating that the surface had superhydrophilic properties.

Energy-dispersive X-ray spectroscopy (EDX) was performed to determine the elemental composition of the prepared substrate. Figure 4d, e illustrates the EDX results obtained for the HS-SERS substrate at an excitation voltage of 10 kV and an excitation time of 50 s. Theoretically, the superhydrophobic surface should comprise a PDMS material fabricated using FLIFT with a molecular formula of (C_2_H_6_OSi)*n* and an elemental composition ratio of C:O:Si = 2:1:1, whereas the superhydrophilic-pattern regions should only contain Si after the removal of the PDMS material using a laser to expose the hydrophilic silicon structure. Figure 4d shows the EDX results obtained for the superhydrophobic surface of the HS-SERS substrate, denoted by the area indicated by the red arrow in Fig. 4a. As revealed by the figure, the surface contained three elements, namely, C (56.21%), O (20.65%), and Si (23.14%), which is consistent with the theoretical results and demonstrates that the area was composed of PDMS. Figure 4e shows the EDX results obtained for the superhydrophilic pattern regions of the HS-SERS substrate, denoted by the area indicated by the black arrow in Fig. 4a. As revealed by the figure, the area contained two elements, namely, O (29.06%) and Si (70.94%). A possible reason for the discrepancy between this result and the theoretical results is that the laser oxidized part of the silicon substrate during the ablation of the single-crystal silicon, which resulted in the incorporation of O.

### The controllable and stable concentration result of the droplet

**Fig. 5:**
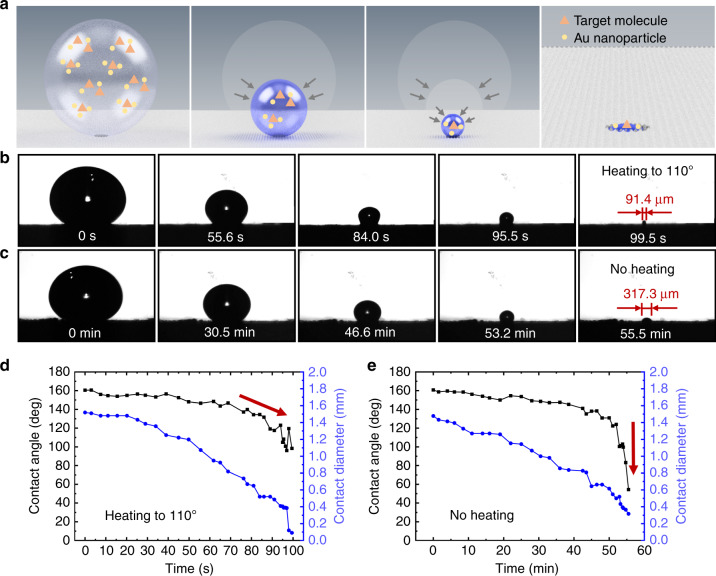
The effect of temperature on the concentration process of droplets. **a** Schematic of the concentration process showing the droplet containing the target molecules and Au nanoparticles. The color of the droplet changes from light to deep; orange–red triangles represent the target molecules, and yellow circles represent Au nanoparticles. **b** Lateral images of the concentration process of the droplet on the HS-SERS surface with a diameter of 100 μm and heated to 110 °C. The droplet volume was 20 μL, and the final contact diameter of the droplet was 91.4 μm at 99.5 s. **c** Lateral images of the concentration process of the droplet on the HS-SERS surface with a diameter of 100 μm under no heating conditions. The final contact diameter of the droplet was 317.3 μm at 55.5 min. **d** Changes in contact angle and contact diameter over time during droplet concentration in **b**. **e** Changes in contact angle and contact diameter over time during droplet concentration in **c**

To provide the required surface plasmon resonance for HS-SERS, precious metal Au nanoparticles with a diameter of 40 nm were mixed with the target molecules and concentrated together on the substrate, which was heated at a temperature of 110 °C. Figure 5a presents a schematic of the droplet containing the target molecules and Au nanoparticles for the concentration process; the orange–red triangles represent the target molecules, and the yellow circles represent the Au nanoparticles. When the HS-SERS substrate was heated, the portion of the droplet that contacted the substrate evaporated to form a thin water vapor film. The water vapor film maintained the droplets in a spherical state to enhance their stability; thus, the droplets did not collapse due to the wet transition during the concentration process. Concurrently, the presence of the superhydrophilic pattern captured the droplets and concentrated them in the designated areas, thereby preventing them from rolling off the substrate. Finally, the target molecules and Au nanoparticles were all concentrated in the superhydrophilic pattern. At the same time, heating can accelerate droplet evaporation, considerably shorten the droplet concentration time, and improve the detection efficiency of the HS-SERS substrate. Because the evaporation environment temperature is 110°, the heated HS-SERS substrate may be more suitable for detecting molecules with thermal stability, such as R6G, quinoline, and other organic compound molecules.

To examine these phenomena, the droplet concentration process on the patterned superhydrophilic substrate with a diameter of 100 μm was investigated, and the corresponding lateral images are presented in Fig. 5b, c. The former HS-SERS substrate was heated to 110 °C (Fig. 5b), and the latter was not heated (Fig. 5c). Under heating conditions, a 20-μL droplet was completely concentrated into the superhydrophilic pattern. Throughout the concentration process, the shape of the droplet remained spherical. The final contact diameter and area of the droplet were 91.4 μm and 0.013 mm^2^ at 99.5 s under the heating conditions. The area decreased by 12.1 times and the evaporation efficiency increased by 33.4 times compared with that of the unheated droplet, and the final contact diameter and area were 317.3 μm and 0.157 mm^2^ at 55.5 min. Figure 5d, e shows the changes in the contact angle and contact diameter with time under heating and nonheating conditions, respectively, and the HS-SERS substrate used under both conditions had a pattern diameter of 100 μm. During the concentration process under the heating conditions, the contact diameter of the droplet was continuously reduced until the droplet was completely concentrated without loss into the superhydrophilic pattern, and the contact angle of the droplet remained at a high value above 140° at 79 s. Then, the angle changed slowly, as denoted by the red arrow in Fig. 5d. Finally, the contact angle remained greater than 100°, and the droplet remained spherical. Under the no heating conditions, as shown in Fig. 5e, the changes in the contact diameter and contact angle in the previous period were the same as those under the heating conditions. However, the droplet suddenly collapsed; the contact angle dropped sharply at 54 min, as indicated by the red arrow in Fig. 5e. The final droplet concentration was 317.3 μm. The entire concentration process lasted for 55.5 min.

**Fig. 6:**
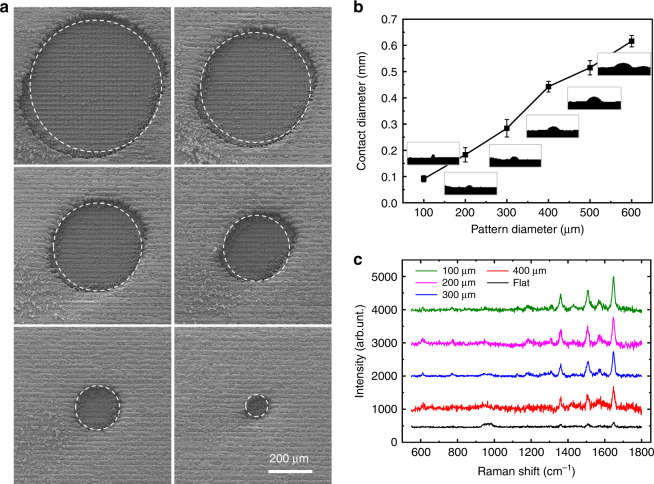
The effect of the size of the superhydrophilic pattern on the concentration process of droplets. **a** SEM images of patterned superhydrophilic surfaces with different diameters, which from left to right are 600, 500, 400, 300, 200, and 100 μm. **b** Final concentrated diameter for superhydrophilic patterns with different diameters. **c** Raman spectra of a 10^−10^ M R6G solution on surfaces with different superhydrophilic pattern diameters

The ability to concentrate the droplet into the superhydrophilic pattern without loss can enable regulation of the droplet concentration by changing the area of the superhydrophilic pattern. To test this hypothesis, superhydrophilic patterns with different diameters, namely, 600, 500, 400, 300, 200, and 100 μm (Fig. 6a), were used to explore the droplet concentration. Figure 6b illustrates the final concentrated diameters for the patterned superhydrophilic surfaces with different sizes, and the data were measured three times. As the diameter of the superhydrophilic pattern decreased, the final concentration diameter decreased, and the size was close to that of the pattern. The results obtained in the three experiments revealed the same trend, indicating the good repeatability of the HS-SERS substrates and providing the basis for accurately controlling the size of the concentrated areas.

To compare the effect of different superhydrophilic pattern areas on the HS-SERS substrate detection intensity, we used an R6G solution, which is a typical dye widely used in biotechnology, with a concentration of 10^−10^ M as the target molecule. We used a laser with a wavelength of 532 nm and a power of 0.05 mW to excite the sample; the integration time was 10 s with three accumulation times. The Raman spectra observed for the 10^−10^ M R6G solution on patterned superhydrophilic substrates with different diameters—including 400, 300, 200, and 100 μm—and untreated silicon substrates are shown in Fig. 6c. We compared the peaks of the HS-SERS spectra at 1650 cm^−1^ and found that the peak intensity increased as the diameter of the superhydrophilic pattern decreased, which demonstrates that the area of the superhydrophilic pattern can effectively regulate the concentrated droplet area, thereby enhancing the detection sensitivity of the HS-SERS substrates.

### The detection limit

**Fig. 7:**
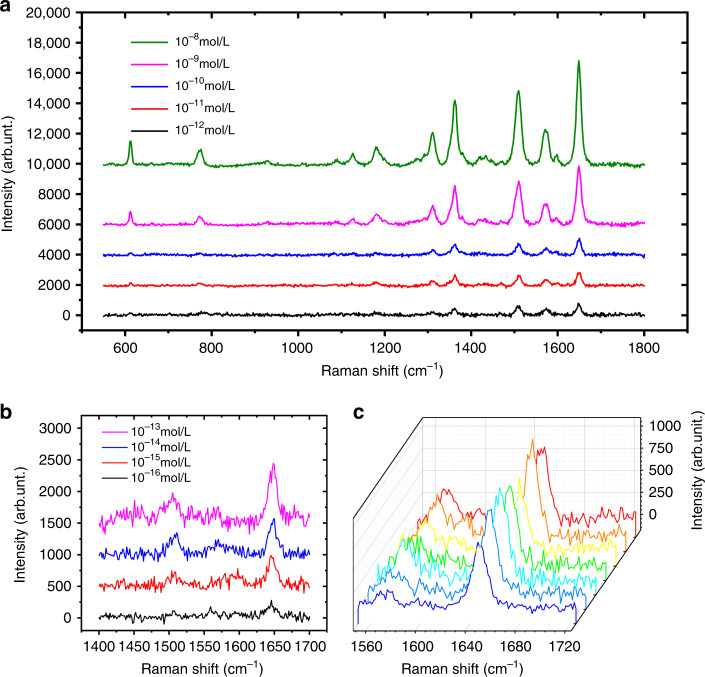
Raman scattering spectra of solutions with different concentrations. **a** Raman spectra of 10^−8^ M–10^−12^ M R6G solutions on the HS-SERS substrate with a pattern diameter of 100 μm. **b** Raman spectra of 10^−13^ M–10^−16^ M R6G solutions on the HS-SERS substrate with a pattern diameter of 100 μm. **c** Raman spectra of seven random points on the HS-SERS substrate with a pattern diameter of 300 μm with the 10^−10^ M R6G solution

The HS-SERS substrate with a pattern diameter of 100 μm was used to test the detection limit of the substrate, and the concentration of the R6G solution used varied from 10^−8^ to 10^−16^ M. Characteristic Raman peaks were observed at 611, 776, 1180, 1360, 1509, and 1650 cm^−1^, and the most intense peak occurred at 1650 cm^−1^, which we chose as the comparison signal. As the concentration of the R6G solution gradually decreased, the Raman peak intensity decreased at 1650 cm^−1^. When the concentration of the R6G solution was 10^−16^ M, the Raman peak could still be measured. Moreover, when the concentration of the R6G solution decreased to 10^−17^ M, the Raman peak intensity at 1650 cm^−1^ was too weak to be captured, as shown in Fig. 7a, b. We randomly selected seven points on the HS-SERS substrate with a pattern diameter of 300 μm to measure the Raman signal of the R6G solution with a concentration of 10^−10^ M. The Raman peak intensity at 1650 cm^−1^ is shown in Fig. 7c, which proves the uniformity of the HS-SERS substrate.

## Conclusion

In conclusion, this paper proposed a simple method for preparing a controllable and stable HS-SERS substrate by a femtosecond laser for ultratrace detection. The method used directly achieves the combination of two surfaces with extreme wettability by a femtosecond laser without additional surface modification. The fabrication process involves only two steps in situ: first, PDMS was sputtered onto the surface of silicon through FLIFT to form a periodic superhydrophobic structure. Subsequently, a femtosecond laser was applied to selectively remove the superhydrophobic structure while ablating the underlying silicon substrate to form a superhydrophilic pattern. We mixed the target molecules and Au nanoparticles and then concentrated them into the pattern through heating to achieve ultratrace detection. The HS-SERS substrate achieved a final contact area of 0.013 mm^2^, 12.1 times less than the unheated case. The reduction in the contact area led to a detection limit concentration as low as 10^−16^ M for the R6G solution. In addition, the HS-SERS substrate accurately controlled the size of the concentrated areas through the superhydrophilic pattern because of the favorable repeatability of the droplet concentration results. The latter was never discussed in SW-SERS substrates and offers the possibility of quantitative SERS detection. In addition, the preparation method is flexible and has the potential for fluid mixing, fluid transport, and biochemical sensors.

## Supplementary information

The supplementary material of “Hybrid superhydrophilic-superhydrophobic micro/nanostructures fabricated by femtosecond laser-induced forward transfer for sub-femtomolar Raman detection”
