# Supplementary material for: Hybrid superhydrophilic–superhydrophobic micro/nanostructures fabricated by femtosecond laser-induced forward transfer for sub-femtomolar Raman detection
Source: Microsyst Nanoeng. 2019 Sep 23;5:48. doi: 10.1038/s41378-019-0090-1 (PMC6799889; doi:10.1038/s41378-019-0090-1)
Supplement: Supplementary file 1 — The supplementary material of “Hybrid superhydrophilic-superhydrophobic micro/nanostructures fabricated by femtosecond laser-induced forward transfer for sub-femtomolar Raman detection” [file 41378_2019_90_MOESM1_ESM.docx]

**Supplementary Information**

**Hybrid superhydrophilic-superhydrophobic micro/nanostructures fabricated by femtosecond laser-induced forward transfer for sub-femtomolar Raman detection**

Xiaodan Ma^1^, Lan Jiang^*1^, Xiaowei Li^1^, Bohong Li^1^, Ji Huang^1^, Jiaxing Sun^1^, Zhi Wang^1^, Zhijie Xu^1^, Liangti Qu^2^, Yongfeng Lu^3^, Tianhong Cui^4^


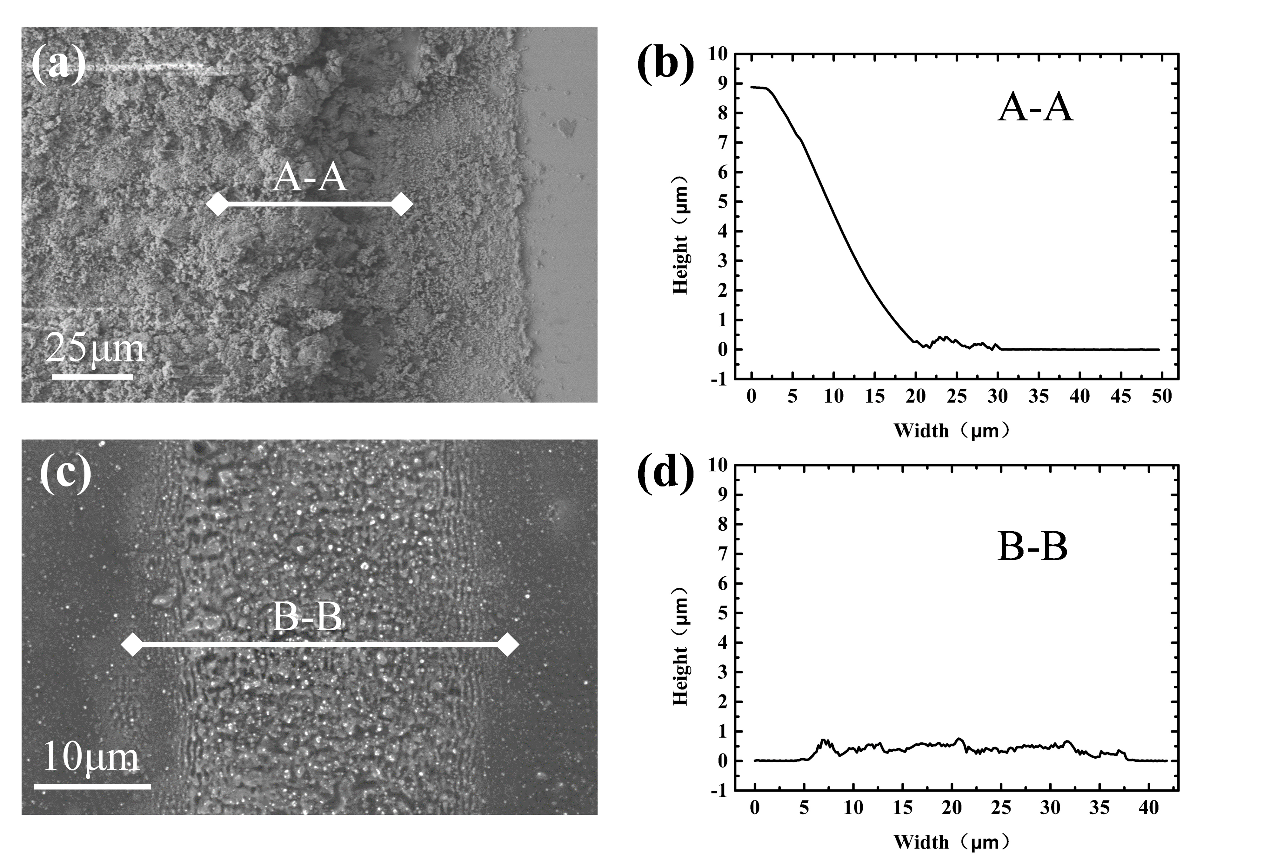


Figure S1. (a) SEM images of the edge morphologies of the Superhydrophobic structure at a laser power of 25 mW and a scanning pitch of 20 μm. (b) AFM contour curve at A-A in (a), Superhydrophobic structure with a height of 9 μm. (c) SEM images of a single-line scanning of femtosecond laser at a laser power of 25 mW. (d) AFM contour curve at B-B in (c), Superhydrophobic structure with a height of 0.8 μm.


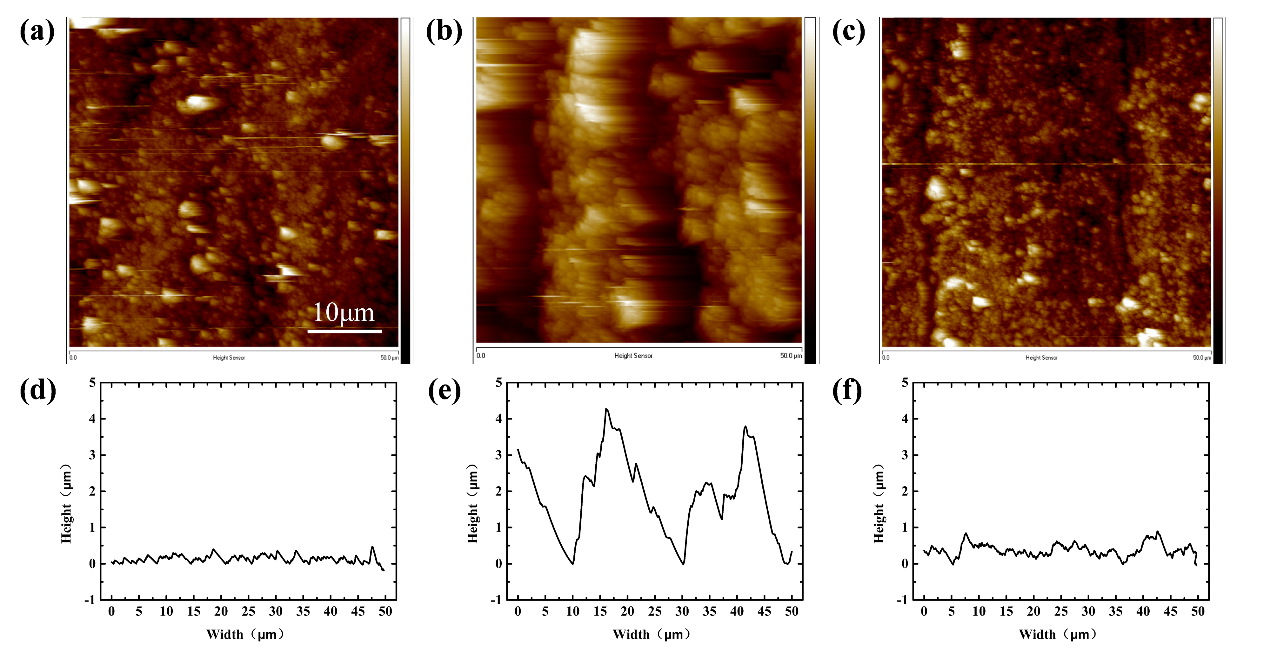


Figure S2. AFM scanning results at different laser scanning pitch and a laser power of 25 mW: (a) 10 μm, (b) 20 μm, (c) 30 μm, AFM contour curve at different laser scanning pitch and a laser power of 25 mW: (e) 10 μm, (f) 20 μm, (g) 30 μm.
